# Deep transfer learning and explainable AI framework for autism spectrum disorder detection across multiple datasets

**DOI:** 10.3389/fneur.2025.1617446

**Published:** 2026-01-13

**Authors:** Shtwai Alsubai

**Affiliations:** 1 Department of Computer Science, College of Computer Engineering and Sciences, Prince Sattam bin Abdulaziz University, Al-Kharj, Saudi Arabia; 2 King Salman Center for Disability Research, Riyadh, Saudi Arabia

**Keywords:** ASD, cross-dataset validation, deep neural networks, explainable AI, healthcare, transfer learning

## Abstract

**Introduction:**

This paper presents a transfer learning approach for Autism Spectrum Disorder (ASD) detection using Deep Neural Networks (DNN) across three distinct datasets.

**Methods:**

A baseline was established by training multiple machine learning and deep learning models on a toddler ASD screening dataset from Saudi Arabia, augmented with the Synthetic Minority Oversampling Technique (SMOTE) to address class imbalance. The DNN architecture featured regularization and dropout layers. The trained model was then leveraged by transferring learned knowledge to two additional ASD datasets. Model performance was analyzed through standard metrics and explainable AI techniques.

**Results:**

The DNN architecture outperformed other models (i.e., LSTM and Attention LSTM). Transfer learning demonstrated improved performance with limited training data. Explainable AI techniques provided insights into key features for ASD classification across different populations.

**Discussion:**

Results indicate the efficacy of transfer learning for cross-dataset ASD classification, suggesting the presence of common behavioral indicators despite demographic and data collection differences.

## 1 Introduction

Autism Spectrum Disorder (ASD) exists as a complex neuro-developmental condition where people show continuous social communication problems with relationship difficulties in addition to repetitive behaviors (1, 2). Representing autism symptoms in different ways among affected people has led to the spectrum description, which results in a highly complicated diagnostic framework (3). Early diagnosis, together with prompt action, plays a crucial role in children's development by enhancing their social capabilities, advancing their cognitive abilities, and improving their behavioral competence (4, 5). The existing diagnostic frameworks rely too heavily on trained clinicians who conduct subjective behavioral assessments. This method requires significant time and resources, yet yields inconsistent results across evaluators working in different clinical environments.

Machine learning (ML) and DL approaches have shown promise as solutions to address the inherent challenges in ASD diagnosis (6–9). Modern techniques are effective at detecting subtle patterns in behavioral, neuroimaging, and genetic data that human experts might not readily recognize (10). Large-scale, well-annotated datasets represent the primary challenge in ML applications for ASD diagnosis (11). The obtainment of proper datasets faces various barriers, including restrictions from privacy concerns combined with ethical challenges and complexities of diagnosis, alongside rare ASD occurrences in the broader population. The existing datasets exhibit strong imbalances, with significantly more non-ASD control examples than ASD cases, leading to algorithmic bias and unreliable predictive models (12). The major impediment in the field stems from the diverse characteristics of datasets. The multiple data sources differ in how they present features alongside collection approaches, population composition, and diagnostic regulations, leading to inevitable difficulties in developing a single model application for all. The lack of standardization poses a significant obstacle to developing detection tools with large-scale potential for various real-world population groups.

This research introduces a deep learning framework for ASD Classification. It employs transfer learning across multiple datasets to address the limitations of current methods. By using transfer learning techniques, models can extract knowledge from related tasks or domains, enabling them to adapt to new, smaller, or more domain-specific tasks. The study examines the use of pre-trained models on large, general-purpose medical datasets and on datasets specifically related to ASD, aiming to identify patterns associated with this disorder. It incorporates sequential fine-tuning across various ASD-specific datasets. This approach not only mitigates issues related to data scarcity and class imbalance but also enables the model to acquire domain-general knowledge, ultimately enhancing its generalization capabilities.

Despite advances in deep learning-based ASD recognition, many existing models struggle to generalize across diverse datasets, limiting their clinical applicability. The primary research question is: Can a deep transfer learning model equipped with explainable AI improve cross-dataset performance and interpretability in ASD Classification? The research problem aims to develop a high-performance, explainable model that is scalable, reliable, and adaptable to diverse datasets for diagnostic purposes in real-world healthcare settings.

Section 2 provides the previous work on this domain. Section 3 describes the datasets used in this research, whereas Section 4 focuses on the methods and models employed. Section 5 presents the results obtained. Section 6 discusses and summarizes the results and benefits gained from the research. Finally, Section 7 concludes the paper and provides future directions for the research.

## 2 Related work

The recent findings have discussed EEG-based methods for the assessment and classification of ASD. In the study (13), the authors have used EEG data (in multi-channel) and Slope Entropy (SlopEn) features to measure ASD severity and classified the patients as mild, moderate and severe using the SVM and the Random Forest classifier with classification accuracy of 98.67 and 97.89, respectively. In another study (14), which used pre-trained deep Convolutional Neural Network (CNN) along with hybrid machine learning classifiers (SqueezeNet + SVM, KNN and DT) to classify ASD severity and differentiate between age-matched controls, maximum accuracy of up to 87.8 percent was achieved through the combination of hybrid models, which can be illustrated in the following way: These publications show that the EEG-based feature extraction, transfer learning, and hybrid modeling have been effective in ASD Classification. Based on these strategies, our work will build on prior studies by combining multi-dataset transfer learning with explainable AI methods to achieve cross-population generalization and enhance the interpretability of model decisions. Similarly, Authors in (15) explored ASD diagnosis using multiple machine learning techniques on the AQ-10-Adult dataset. Their study examined various classifiers across different scenarios, demonstrating the feasibility of automating autism diagnostic tools using machine learning. They achieved promising classification accuracy by applying traditional machine learning algorithms to questionnaire-based screening data, highlighting the potential of computational methods to support clinical decision-making in ASD diagnosis.

Authors in (16) presented a vital discussion on ASD early detection and developed a CNN-based transfer-learning model, following the AlexNet architecture (ASDDTLA), to evaluate facial attributes extracted from publicly available facial images for classifying autistic versus non-autistic children. Research findings indicate that the proposed ASDDTLA model achieves a detection success rate of 87.7% and outperforms other renowned ASD Classification models, highlighting its practical potential for early clinical application. This study (17) proposed a novel strategy for early detection of ASD in young children by applying machine learning to brain images within an Internet of Things (IoT) framework. The research employs CNNs and transfer-learning algorithms to process combined data from the Autism Brain Imaging Data Exchange (ABIDE I and ABIDE II). The model incorporates pretrained network components, which the team further fine-tunes using autism-specific brain scan information to enhance diagnostic precision. The study results show that the improved CNN model achieved an accuracy of 81.56%, surpassing traditional approaches that operated only on the ABIDE I database. The detection performance of ASD in IoT environments demonstrates promising potential with transfer learning.

Authors in (18) investigated two classifiers, MMEC and MSEC, that utilize transfer learning to improve the efficiency of ASD Classification from functional magnetic resonance imaging (fMRI) data. The MMEC system integrates Inception V3, ResNet50, MobileNet, and DenseNet deep learning models, while MSEC combines base classifiers obtained from different datasets. The evaluation framework comprises ensemble averaging, weighted averaging, and stacking methods for model assessment. Research results show that MMEC with stacking delivers optimal outcomes by improving MSEC performance, boosting accuracy by 3.25%–97.78% across multilocation datasets. Deep learning (19) and transfer-learning-based prediction methods for ASD are the primary focus of this study. To predict autism, the authors use pre-trained deep learning models and fine-tune them on an appropriate dataset. Training the proposed approach requires the use of extracted input features alongside behavioral or physiological datasets.

Recent studies have been finding it more useful to incorporate multimodal information to classify ASD, beyond questionnaire answers, into a range of other sources, such as eye-tracking, facial expression, speech, and neuroimaging, as introduced in the authors of (20). Indicatively, hybrid deep learning models that integrate visual, speech, and facial information are highly accurate in ASD diagnosis when using eye-tracking data, suggesting that multimodal fusion can provide a more comprehensive behavioral characterization. In the same paper, the authors applied multimodal frameworks that combine structural MRI and behavioral phenotypes to provide explainable latent representations that improve classification performance and intercohort strength (21). The systematic mapping of AI applications that the authors of (22) focused on has also highlighted the use of voice, movement, and visual attention features in ASD Classification, underscoring the diverse modalities that can supplement traditional screening tools. In the study in question, the authors (23) mentioned that one of the primary problems in ASD machine learning research is the heterogeneity of the datasets, such as the ability to vary in terms of sample size, demographic bias, and instrument specificity, which leads to the limitation of the generalizability and cross-cohort effectiveness. In another study (24), similar to this one, the authors state that questionnaire-based screening tools, although practical, differ in structure and are more diagnostic, making it challenging to compare tools or apply them directly to another model. For example, statistical models trained on one age group or screening type may not be transferable to another without close harmonization of feature spaces, the primary motivation for transfer learning research.

Authors in (25) presented an original method for detecting ASD at an early stage through the analysis of emotional facial features. The system combines transfer-learning features derived from facial images with handcrafted image features, using VGG16, ResNet, and Inception models, along with HOG, LBP, SIFT, and PHASH descriptors. A combination of machine learning classifiers, including Random Forest, KNN, Decision Tree, SVM, and Logistic Regression, is used to process the merged feature vectors. A performance evaluation of these classifiers is conducted on three datasets to determine the optimal combination for accurate ASD diagnosis. The research (26) introduces a framework to advance ASD diagnosis through resting-state functional Magnetic Resonance Imaging (rs-fMRI) data. The research targets the heterogeneity problem in multi-site rs-fMRI datasets, which undermines current ASD Classification systems. The proposed approach performs domain adaptation on heterogeneous fMRI data before applying transfer learning for ASD prediction, thereby boosting model generalization. A study using a custom deep CNN analyzed rs-fMRI data and achieved high diagnostic metrics, including 99.39% accuracy, 98.80% recall, 99.8% precision, and a 99.32% F1 score, according to one report. This demonstrates that fMRI data analyzed with advanced deep learning methods can provide substantial improvements in ASD Classification.

Past research on ASD Classification has focused mainly on single-dataset-based learning pipelines, making it hard to generalize to different populations. They achieved good intra-dataset accuracy but failed to assess transferability across different demographic groups. Our work, in turn, proposes a transfer-learning model that leverages information acquired from one ASD group (e.g., toddlers) and applies it to other groups (children and adults). In addition, although explainable AI has been used in other studies on neurological disorders, its use together with multi-dataset ASD classification is not well studied. In our framework, we integrate XAI modules, such as Grad-CAM and SHAP, into the deep network training pipeline, thereby providing interpretable feature attribution maps that directly relate to clinically meaningful behavioral indicators. This two-fold interest in transferability and explainability makes our method stand out from previous ASD detectors, which have focused only on accuracy, not interpretability or cross-population validation.

## 3 Dataset selection

This study utilizes three distinct ASD-related datasets, which are discussed in this section.

### 3.1 Dataset 1: Saudi Arabia toddlers

The research dataset consists of screening information for Saudi Arabian toddlers,^1^ aged 12–36 months. Our transfer learning framework uses data collected from participants via an online Google Forms questionnaire, which served as the source domain. The questionnaire incorporated the Arabic translation of the Q-CHAT-10 (Quantitative Checklist for Autism in Toddlers) to screen for autism spectrum disorder in early childhood. A total of 10 behavioral screening questions were included in the survey, and respondents were evaluated using a 5-point Likert scale to distinguish typical from atypical behaviors. The evaluation questions directly assess signs of ASD through social cue recognition, gesturing, social engagement behaviors, and imaginative play. The dataset contains behavioral questions alongside demographic data points, including the toddler's age in months, gender, residential region, family history of ASD, and respondent type (parent or guardian). The Q-CHAT-10 protocol scored 1 point for matching categories C, D, or E for questions 1-9, and 1 point for responses in categories A, B, or C for question 10. The screening score was calculated by summing all positive responses to the ten questions. Additional ASD traits were identified when a score exceeded the threshold, yielding a binary output indicating potential ASD characteristics. At the same time, 0 showed the absence of such traits. The data features used to analyze the Saudi Arabian population are presented in Table 1.

**Table 1:** Feature descriptions for Saudi Arabia toddlers dataset.

**Feature**	**Description**
A1-A10	Binary-coded responses to Q-CHAT-10 screening questions.
Age	Toddler's age in months.
Gender	Categorical variable (Male/Female).
Region	Categorical variable indicating the geographic region within Saudi Arabia.
Screening score	Numerical value ranging from 0 to 10.
Family history of ASD	Boolean indicator (Yes/No).
Test administrator	Categorical feature denoting who completed the questionnaire (e.g., parent).
Class	Target variable representing the ASD trait classification.

### 3.2 Dataset 2: SciDB dataset

The SciDB dataset^2^ functions as one of the target domains dedicated to our transfer learning framework in this research. The dataset includes ASD symptom data collected from toddlers using the Modified Checklist for Autism in Toddlers, Revised (M-CHAT-R). A diverse group of children aged 3–15 years filled out the symptomatic data for ASD using the Modified Checklist for Autism in Toddlers Revised (M-CHAT-R). Data collection took place in North Cairo Governorate, Egypt. It involved various organizations, including the Ain Shams Center for Special Needs Care, the Egyptian Autism Society, and the Resala Charity Organization. Direct parent interviews were also conducted via WhatsApp and Facebook. The primary purpose of this dataset is to support research and machine learning applications for Arabic-speaking populations while providing real-world ASD data collected in its native cultural environment. A single M-CHAT-R screening entry in this dataset corresponds to 13 binary features (Q1–Q13) derived from parents' responses to the recognized autism detection questions. The dataset contains demographic data about children together with their clinical history, including age, diagnosis type and gender, alongside a binary ASD trait indicator. The extensive diagnostic backgrounds and age-range diversity in the SciDB dataset lead to label inconsistencies and variable features, thereby providing essential assessment capabilities for deep learning model robustness in realistic clinical settings. This database serves as a target domain for transfer learning, enabling evaluation of models trained on structured Saudi toddler data and their ability to generalize across datasets with different demographic distributions and higher data noise.

### 3.3 Dataset 3: autism in adults

The third dataset in this study is from a Kaggle competition [https://www.kaggle.com/competitions/autismdiagnosis/data] on ASD recognition and serves as an additional target dataset for our transfer-learning implementation. A wide set of features, including clinical data and demographic information, has been collected from diverse patients to create this dataset on ASD screening. The records contain numerous data fields, including patient identifiers under ID and scores ranging from A1_Score to A10_Score, derived from the Autism Spectrum Quotient screening tool, as well as test results. The assessment of ASD traits relies heavily on these scores, as they serve as primary indicators for examining ASD trait likelihood in patients. The dataset includes information on patients' age, gender, and ethnic background, providing vital demographic details. The dataset comprises information on jaundice at birth (jaundice), autism family background (autism), the patient's country of residence (country_of_res), and prior exposure to ASD screening (used_app_before). The provided variables enhance the dataset by integrating clinical records and sociodemographic factors that influence diagnostic outcomes. The target variable within the Class/ASD group classifies patient data into two categories: 0 signifies no ASD traits, and 1 signifies potential ASD characteristics. The dataset includes age_desc for patient age descriptions and relation for the tester's family connections. The data enhances our understanding and increases the dataset's complexity as reporting methods become more variable. The combination of screening tool scores, demographic features, and medical background creates an optimal database framework for transfer learning applications. Preprocessing data and uniting features becomes difficult because this dataset contains a wide range of features with varying levels of measurement detail.

## 4 Proposed framework

**Figure 1:**
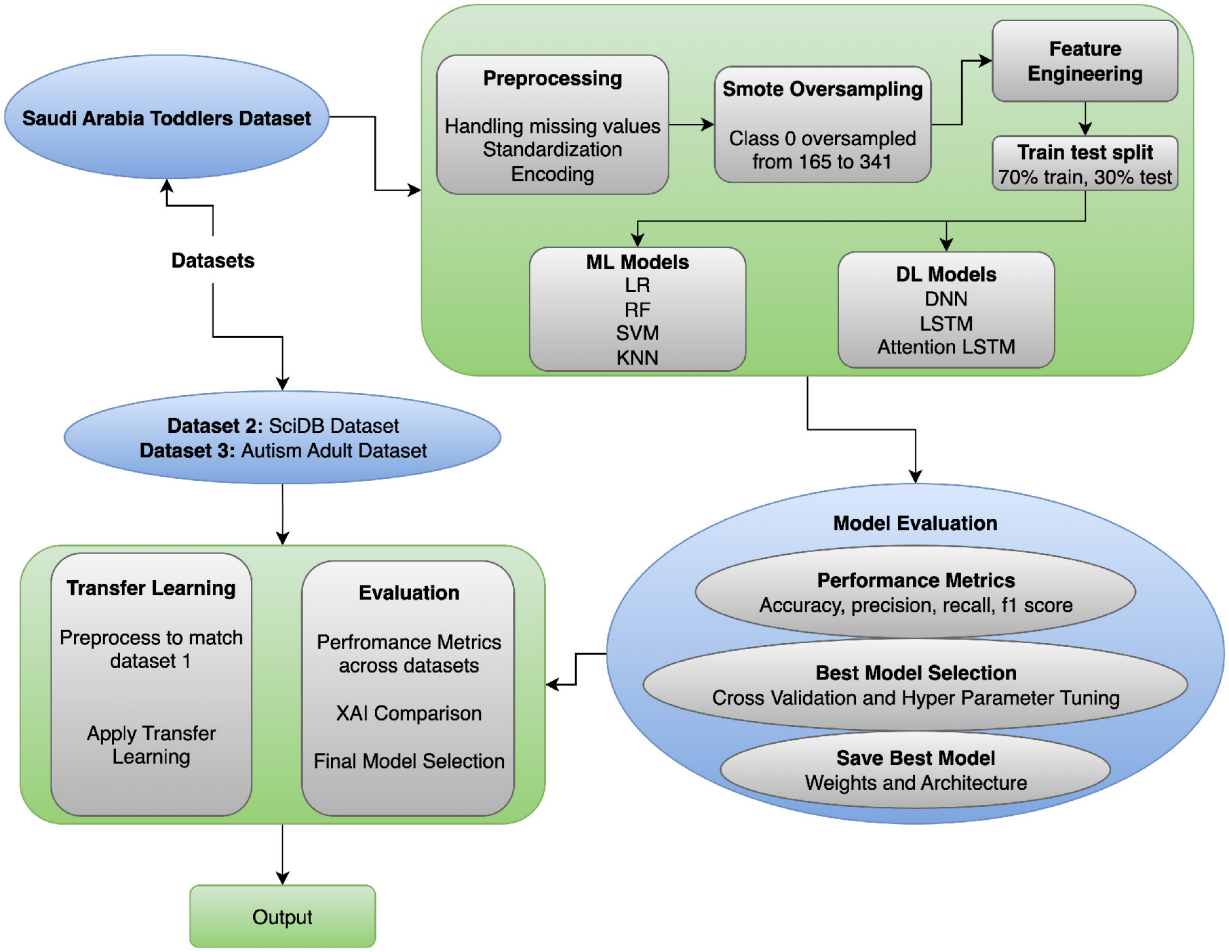
ASD classification approach flow diagram.

This section focuses on the proposed methodology for ASD Classification using transfer learning. This section explains the preprocessing steps for the datasets, the model architectures, and both traditional machine-learning and advanced deep-learning models. It also describes the transfer-learning approach used with the model and presents the ASD Classification framework. The proposed workflow is shown in Figure 1 and incorporates preprocessing, hybrid modeling, transfer learning, and explainable evaluation to achieve robust ASD Classification. Data are typically cleaned and encoded using SMOTE, and feature alignment is performed to ensure inter-dataset compatibility. Deep learning models (e.g., DNN, LSTM, Attention LSTM) are trained, and transfer learning enables adaptation of knowledge to demographic contexts. The interpretability of explainable AI (Grad-CAM, SHAP) makes the framework generalizable and clinically transparent, unlike previous single-dataset, non-explainable studies (see Algorithm 1).

Algorithm 1 **ASD classification framework with transfer learning.**


1: **Input:** Dataset_1_, Dataset_2_, Dataset_3_
2: **Output:** Final ASD Classification model
3: {——- Train Baseline Models ——-}
4: (*X, y*)←LoadData(Dataset_1_)
5: (*X*_bal_, *y*_bal_)←SMOTE(*X, y*)
6: (*X*_train_, *X*_test_, *y*_train_, *y*_test_)←TrainTestSplit(*X*_bal_, *y*_bal_)
7: models←{DNN, LSTM, Attention-LSTM}
8: **for** each model in models **do**
9:    model.fit(*X*_train_, *y*_train_)
10:    metrics←ComputeMetrics(*y*_test_, model.predict(*X*_test_))
11:    results[*model*]←metrics
12: **end for**
13: bestModel←SelectBestModel(results)
14: {——- Apply Transfer Learning ——-}
15: transferModel_2_ ← LoadPretrainedModel(bestModel)
16: transferModel_2_.fit(Dataset_2_)
17: metrics_2_ ← ComputeMetrics(Dataset_2_)
18: transferModel_3_ ← LoadPretrainedModel(bestModel)
19: transferModel_3_.fit(Dataset_3_)
20: metrics_3_ ← ComputeMetrics(Dataset_3_)
21: {——- Compare and Evaluate ——-}
22: finalModel←SelectBestModel(bestModel, metrics_2_, metrics_3_)
23: **return** finalModel

### 4.1 Data preprocessing

The research preprocessing framework was designed to address common problems in healthcare data, including data gaps, class imbalance, and inconsistent feature measurements. Multiple specialized methods exist to address data-preparation challenges, ensuring data readiness before training operations begin. We use distinct imputation methods for each feature type to handle missing values. The preferred approach for numeric data features is median-based imputation (27). The use of median-based imputation is notable because it protects the data distribution from distortion by outliers during imputation. The translation of the median computation for feature *x* exists in Equation 1.


$$\begin{array}{l} \text{Median}\left(x\right)=\{\begin{array}{cc} x_{\left(\frac{n+1}{2}\right)} & \text{if }n\text{ is odd},\\ \frac{x_{\left(\frac{n}{2}\right)}+x_{\left(\frac{n}{2}+1\right)}}{2} & \text{if }n\text{ is even}, \end{array} \end{array}$$ (1)

The equation uses the number of sorted elements from *x* as parameter *n*. The most frequent category value functions as the imputation method (mode imputation) for categorical features. The most prevalent category in the feature is used to impute any missing values. For a categorical feature x, the mode $\hat{x}$ is calculated from the formula presented in Equation 2.


$$\begin{array}{l} \hat{x}=\text{mode}\left(x\right)=\arg\underset{c\in x}{\max}\text{count}\left(c\right) \end{array}$$ (2)

The *C* contains two components representing both possible categories C and the count value count(c) from category c. We standardize numeric features by rescaling their ranges to equalize their distributions, thereby minimizing the influence of features with wide numeric ranges on the model. Standardization rescales numerical data so that each feature has a median of 0 and a standard deviation of 1. The formula presented in Equation 3 enables standardization of the process.


$$\begin{array}{l} z_{i}=\frac{x_{i}-\mu }{\sigma }, \end{array}$$ (3)

Standardization applies Equation 3 to transform *x*_*i*_ values using μ mean and σ standard deviation of the feature. Distance-based model algorithms require standardization to achieve optimal performance, as they rely on gradient-based optimization or k-nearest neighbors. Each category in a categorical feature is represented as a binary vector via one-hot encoding. When processing one-hot encoding for a categorical feature that contains *k* distinct categories, the method produces *k* binary features, each corresponding to a category. The definition of one-hot encoding specifies the representation for category *c*_*i*_ as in Equation 4.


$$\begin{array}{l} \text{One-hot encoding}\left(x=c_{i}\right)=\left[\begin{array}{c} 0\\ \vdots \\ 1\\ \vdots \\ 0 \end{array}\right], \end{array}$$ (4)

Here, one appears as the only value in the position indicating *c*_*i*_ with the remaining slots filled by zeroes. The Synthetic Minority Over-sampling Technique (SMOTE) produces synthetic data samples for the minority class to balance the dataset. Using SMOTE, the minority class is augmented with synthetic samples generated by interpolating between its current samples. In the synthesis of a new minority class example *x*_*s*_ between two minority class samples *x*_*i*_ and *x*_*j*_, the mathematical representation follows Equation 5.


$$\begin{array}{l} x_{s}=x_{i}+\lambda \cdot \left(x_{j}-x_{i}\right), \end{array}$$ (5)

The random scalar value λ ranges between zero and one for synthesizing test examples from the points *x*_*i*_ and *x*_*j*_ through line approximation. The Function generates examples from minority classes to equalize class proportions, thereby improving the training model's performance, particularly on highly imbalanced datasets. Installing these preprocessing methods, which include median-based and most-frequent imputation, standardization, one-hot encoding, and SMOTE, enables researchers to optimize their data training process. These methods address common problems, such as data gaps, scaling requirements, and class discrepancies, thereby improving machine learning model performance and generalization.

### 4.2 Feature alignment

The datasets employed in the study vary significantly in age groups, screening measures, and data-collection contexts, as ASD assessment is heterogeneous across the lifespan. The sample of dataset 1 addresses the age group of toddlers, 12-36 months, with the assistance of Q-CHAT-10, which concentrates on early developmental and social-communication behaviors. Dataset 2 comprises children aged 3-15 years who were assessed with the M-CHAT-R, with a focus on behavioral risk factors relevant to early childhood screening. In contrast, Dataset 3 is made up of adults who were assessed with the AQ-10, which covers self-reported characteristics that could be related to autism in the later stages of development. These instruments vary in their format, item development and behavioral emphasis and are not clinically interchangeable. Based on this, the study aims to test statistical and representational transferability across age groups, rather than asserting direct clinical equivalence. Rather, the goal is to determine whether learned representations can be statistically transferred between heterogeneous datasets of questionnaires, thereby evaluating whether latent behavioral patterns produced by machine learning models exhibit a transferable structure despite developmental and contextual variation. This difference is clearly recognized as a limitation, and the findings are exploratory rather than clinically prescriptive.

To aid transfer learning between heterogeneous ASD screening datasets, feature alignment was done at the level of representation and not by what is known as semantic correspondence of questions to questions. Because the screening instruments differ in structure and concept, their preprocessing was performed separately, yielding a harmonized numerical feature space for cross-domain learning. Responses to questionnaires were coded as binary or continuous risk variables, and demographic and contextual variables were encoded using one-hot or binary encoding schemes. Imputation of missing values using the median or mode was employed to ensure data robustness in the presence of missing records. Composite features (i.e., total screening scores, ratio of symptoms, or interaction terms where possible) were computed to minimize reliance on dataset-specific item-formulations and to describe higher-level behavioral patterns. All features were then normalized to ensure consistent scaling across datasets. Moreover, feature-importance-based selection was used to retain the most informative attributes in each dataset, thereby reducing dimensionality incompatibility and mitigating noise. This alignment approach is not based on the strict semantic equivalence of individual items in the questionnaire set; rather, it preserves clinically relevant behavioral data and enables evaluation of patterns of latent ASD-related behavioral issues that can be generalized across age groups, populations, and screening measures.

### 4.3 Deep neural network architectures

The study employs a Deep Neural Network (DNN) because this model effectively transforms high-dimensional data into non-linear complex representations (28). The proposed DNN follows Algorithm 2, which integrates representational strength with regularization to reduce overfitting.

Algorithm 2 **DNN model architecture.**


1: **Function** (Initialize deep learning algorithm)
2:    model←Sequential Deep learning Model()
3:    model.add(Dense layer(128,′*relu*′,)
4:    input_shape = inputShape, l1_l2(1*e* − 5, 1*e* − 4))
5:    Dropout(0.4)
6:    model.add(Dense(64,′*relu*′, l1_l2(1*e* − 5, 1*e* − 4)))
7:    Dropout(0.3))
8:    model.add(Dense(32,′*relu*′, l1_l2(1*e* − 5, 1*e* − 4)))
9:    Dropout(0.3))
10:    model.add(Dense(2,′*softmax*′))
11:    model.compile(optimizer = Adam(0.001),
12:    metrics = ['accuracy'])
13: **return** model

The first layer of the DNN architecture comprises an input unit with *d* preprocessed features, as in Equation 6.


$$\begin{array}{l} \text{x}={\left[x_{1},x_{2},\ldots ,x_{d}\right]}^{⊤}\in {\mathbb{R}}^{d} \end{array}$$ (6)

The model has three hidden layers following the input layer, each with 128, 64, and 32 neurons, respectively. All hidden layers employ nonlinear activation functions. Equation 7 shows the mathematical expression of the ReLU activation function, which we apply to the model.


$$\begin{array}{l} \text{ReLU}\left(z\right)=\max\left(0,z\right) \end{array}$$ (7)

The model employs elastic net regularization to mitigate overfitting, along with L1 and L2 regularization. The combined regularization penalty is added to the loss function and is given by Equation 8.


$$\begin{array}{l} \Omega \left(\text{w}\right)=\lambda_{1}\|\text{w}|{|}_{1}+\lambda_{2}\|\text{w}{\|}_{2}^{2} \end{array}$$ (8)

This penalty includes two weights λ_1_ and λ_2_ for L1 and L2 regularization strength control with values of 1e-5 and 1e-4. The deep neural network model incorporates dropout layers applied after each hidden layer, with rates of 0.4, 0.3, and 0.3, respectively. During training, dropout sets the *p* of input units to random values, thus avoiding neuron co-adaptation and controlling overfitting. The final stage comprises a softmax output unit that performs binary classification. The softmax operation uses the output logits *z*_0_ and *z*_1_ to generate class probabilities using the following Equation 9.


$$\begin{array}{l} P\left(y=i|\text{z}\right)=\frac{e^{z_{i}}}{e^{z_{0}}+e^{z_{1}}},\;i\in \left\{0,1\right\} \end{array}$$ (9)

The model optimization uses an Adam optimizer with a learning rate of α = 0.001, combining features from the Adaptive Gradient Algorithm (AdaGrad) and Root Mean Square Propagation (RMSProp). The chosen loss function was categorical cross-entropy, which follows the mathematical expression in Equation 10.


$$\begin{array}{l} L=-\overset{N}{\underset{i=1}{\sum }}\overset{2}{\underset{j=1}{\sum }}y_{ij}\log\left({ŷ}_{ij}\right) \end{array}$$ (10)

The true label *y*_*ij*_ receives prediction values from the predicted probability ŷ_*ij*_ for classification *j* within sample *i*. The specifically designed DNN architecture strikes an appropriate balance between model complexity and regularization, enabling successful learning from scarce, imbalanced data to recognize and detect ASD patterns.

### 4.4 Transfer learning framework

**Figure 2:**
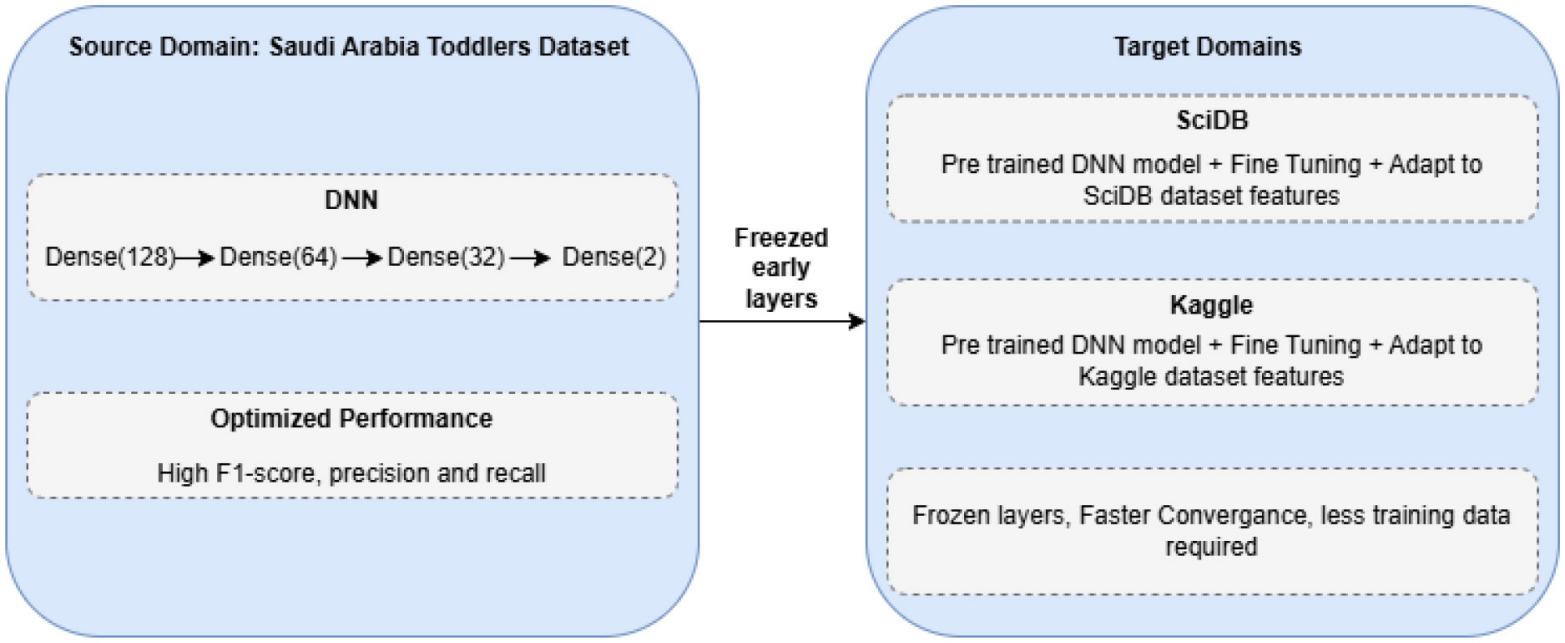
Transfer learning approach for ASD classification.

The primary use of transfer learning allows us to leverage insights from one area (source) to improve performance on a similar task (target) when data are scarce (29). This strategy appears in Figure 2. We begin by training a DNN on data from Saudi Arabian toddlers, which serves as the source domain. The model training continues until it reaches stability, and the system chooses model parameters θ through source domain loss optimization based on Equation 11.


$$\begin{array}{l} \theta^{*}=\arg\underset{\theta }{\min}L_{\text{source}}\left(f\left(\text{x};\theta \right),\text{y}\right) \end{array}$$ (11)

Our approach employs a DNN model *f*(**x**; θ) with Saudi Arabia Toddlers data training to produce optimal parameters θ^*^ through the loss function $L_{source}$. When the model reaches its limit, the best parameter values θ^*^ are saved for later use. The process begins by aligning both target datasets (SciDB and Kaggle ASD) to the format and dimensionality of Dataset 1. After data preparation, the pre-trained model is loaded, and its first two layers, including the input and hidden layers, remain fixed. The initial layers of the network learn basic image characteristics that are generalizable across different datasets. The model trains the last layers to adapt the network to the unique features of each target dataset. Fine-tuning modifies the adaptive layer parameters θ_*t*_ while keeping the fixed layers θ_*f*_ fixed. The process finds the target-domain loss minimum using Equation 12.


$$\begin{array}{l} \theta_{t}^{\prime }=\arg\underset{\theta_{t}}{\min}L_{\text{target}}\left(f\left(\text{x};\theta_{f},\theta_{t}\right),\text{y}\right) \end{array}$$ (12)

Our adapted model undergoes thorough testing using standard performance metrics, including accuracy, precision, recall, F1-score, and AUC, to provide reliable results across all datasets. Our approach enables us to transfer knowledge from extensive source data to improve predictions on target data despite limited sample sizes.

### 4.5 Explainable AI integration

Our deep learning model enhances interpretability by incorporating explainable artificial intelligence (XAI) techniques into its prediction process. Using LIME helps us analyze the impact of features on model results. LIME creates a simple, interpretable model that explains how each feature affects specific predictions. By examining multiple samples and synthesizing their explanations, we can identify essential patterns of features at a broader level. Our method enables us to identify and analyze which features most influence decision-making in each dataset in this study. We enhance human trust in our system by presenting feature weights as bar charts and instance-level explanation graphs to help users understand our deep learning model.

The Explainability AI (XAI) approach to this framework can significantly enhance the interpretability and clinical validity of the classification process. Using LIME and SHAP, the framework identifies which behavioral and demographic factors most strongly influence the prediction outcome. Such visualizations enable clinicians and behavioral professionals to align model reasoning with accepted diagnostic standards, thereby increasing confidence in the model's decisions. Such interpretability allows the gap between data-driven inference and clinical knowledge to be closed, thereby positioning the model as a decision-support instrument rather than a substitute for human expertise.

### 4.6 Assumptions, contributions, and limitations

The proposed framework is based on several key assumptions to ensure methodological consistency and good transferability across datasets. All datasets (Saudi Arabia Toddlers, SCDB, and Autism Adult) are assumed to represent the same features (after standardized preprocessing operations, such as normalization, encoding and class balancing). EEG and behavioral characteristics are considered markers of ASD-related neural and cognitive differences, and the SMOTE implementation is not expected to introduce significant bias. In addition, they assume the ability to transfer learning across datasets, given common latent feature patterns and the selected DL architectures' capacity for inter-domain generalization. The present project provides a single Deep Transfer Learning and Explainable AI system to detect ASD and identify patterns in disparate datasets. It proposes a multi-dataset hybrid model pipeline that combines classical and deep learning models to learn multiple feature representations. Explainable AI methodologies (Grad-CAM and SHAP) also contribute to interpretability, enabling model outputs to be associated with clinically relevant features. All of these contribute to knowledge of cross-domain ASD analysis and facilitate the transparency and transferability of models. Although it has good prospects, it is not without limitations. The model may perform poorly on unseen datasets under different acquisition conditions, necessitating retraining or fine-tuning. Synthetic balancing with SMOTE reliance may not accurately reflect the natural data distribution. Moreover, EEG/behavioral features are the only framework being considered, and multimodal data integration (e.g., MRI, genetic, or speech) should be explored in the future. However, these limitations also offer effective avenues for the framework's development, thereby strengthening its practicality.

### 4.7 Hardware specifications and model hyperparameters

In a bid to maintain reproducibility and transparency, the hardware and implementation environments in this paper are provided in detail. All experiments were conducted in a controlled cloud-based environment using Google Colab Pro. The system was equipped with an NVIDIA Tesla T4 with 16 GB of VRAM, an Intel Xeon processor running at 2.30 GHz, and 27 GB of system memory. Python 3.10, TensorFlow 2.15, and Scikit-learn 1.4 were used as the software environment. In this setup, the mean training time per epoch was different between the architectures: DNN needed about 18–25 seconds per epoch, the LSTM model needed about 32-40 seconds, and the Attention-LSTM needed about 40–55 s, with the bigger computational units of sequential and attention mechanisms. Early stopping was performed with a patience value of 10, with validation loss monitored, and model checkpoints were saved at the highest validation accuracy. The training set was split into a 20% validation set. Categorical cross-entropy was used as the loss function, and accuracy was used as the evaluation metric; all models were trained.

The sampling of hyperparameters was kept consistent across the three models to make sure that the differences in performance are the result of architectural design, rather than the differences in optimisation. A complete summary of the hyperparameter settings is summarized in Table 2. The DNN consisted of three fully connected layers with 128, 64, and 32 units, respectively, each with ReLU activation and dropout rates of 0.4, 0.3, and 0.3. L1 and L2 regularization reduced overfitting with 1 × 10^−5^ and 1 × 10^−4^. The LSTM model used stacked recurrent layers with 128, 64, and 40 units, each followed by dropout and recurrent dropout. The Attention-LSTM architecture expanded the model with Multi-Head Attention with four attention heads and a key dimension of 32, with Layer Normalization and a skip connection. Each model was implemented using the Adam optimiser, with a batch size of 32 and a learning rate of 0.001. The maximum number of training epochs was set to 100, with early stopping (patience = 10) to prevent overfitting. The loss function was categorical cross-entropy, and training was performed with a 20% validation split.

**Table 2:** Model hyperparameters for DNN, LSTM, and Attention-LSTM.

**Component**	**DNN**	**LSTM**	**Attention-LSTM**
Input shape	(features)	(10, 1)	(10, 1)
Hidden layers	128 → 64 → 32	LSTM 128 → LSTM 64 → LSTM 40	LSTM 128 + Multi-Head Attention
Regularization	L1 = 1e-5, L2 = 1e-4	Same	Same
Dropout	0.4, 0.3, 0.3	0.4, 0.3, 0.3	0.4 + 0.3 + 0.3
Attention	-	-	MHA (4 heads, key_dim = 32)
Activation	ReLU	- (LSTM internal gates)	ReLU (dense layers)
Output layer	Softmax (2 units)	Softmax (2 units)	Softmax (2 units)
Optimizer	Adam (0.001)	Adam (0.001)	Adam (0.001)
Loss	Categorical cross-entropy	Same	Same
Batch size	32	32	32
Epochs	100 (with early stopping)	100	100
Early stopping	Patience = 10	Patience = 10	Patience = 10
Checkpoints	Best val-accuracy	Best val-accuracy	Best val-accuracy

The chosen hyperparameters provided a balanced trade-off between model complexity and generalization capability. The consistent use of regularization, dropout, and early stopping ensured stable convergence across all models despite variations in architectural depth. The Attention-LSTM, which is the most computationally intensive due to the attention mechanism, benefited from a GPU-accelerated environment. The hardware setup sufficiently supported training across all architectures without memory bottlenecks, and the detailed hyperparameter specification enables other researchers to reproduce the experimental settings with fidelity.

## 5 Experimental results

The experimental and analysis section explains the performance of each classifier applied in this study. Various DL models were evaluated on multiple datasets to assess their effectiveness. The performance evaluation was based on accuracy, precision, recall, and F1-score. Furthermore, transfer learning was employed to improve model generalization, and Explainable AI (XAI) techniques were applied to interpret model decisions.

### 5.1 Model performance

The LSTM and its attention-based update demonstrated below-average performance, achieving precisely 90% accuracy across all metrics (see Table 3). Although LSTMs can capture temporal patterns, this dataset format does not confer significant advantages on recurrent models. The additional attention mechanisms in the model did not yield performance improvements over the standard LSTM. The DNN model achieved the highest performance, with a maximum validation accuracy of 0.9908 during training, prompting researchers to select it as the base model for future exploration.

**Table 3:** Deep learning model performance on dataset 1.

**Model**	**Precision**	**Recall**	**F1-score**	**Accuracy**
DNN	0.96	0.96	0.96	0.96
LSTM	0.90	0.90	0.90	0.90
Attention LSTM	0.90	0.90	0.90	0.90

As the paired t-test values (Table 4) suggest, the DNN model is significantly better than the LSTM and Attention-LSTM models in terms of the classification accuracy. The two architectures (LSTM and Attention-LSTM) are not statistically distinguishable, indicating similar performance. These results demonstrate that the DNN achieves the best performance among the three models on the existing dataset.

**Table 4:** Paired *t*-test results comparing DNN, LSTM, and Attention-LSTM models.

**Model pair**	***t*-statistic**	***p*-value**	**Significance**
DNN vs LSTM	17.2091	0.0000	Significant
DNN vs Attention-LSTM	11.8017	0.0000	Significant
LSTM vs Attention-LSTM	1.6236	0.1389	Not significant

**Figure 3:**
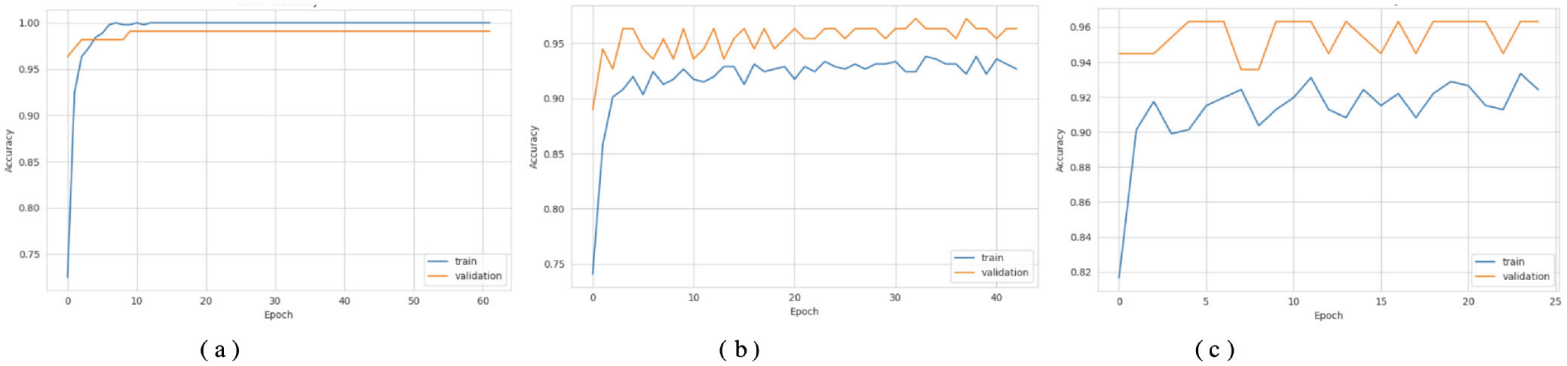
Accuracy curves of the classifiers. **(a)** DNN. **(b)** LSTM. **(c)** Attention LSTM.

**Figure 4:**
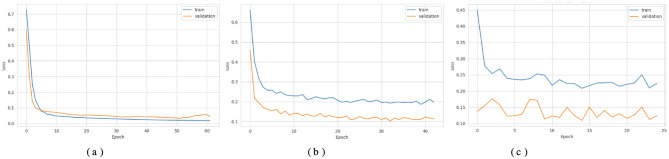
Loss curves of the classifiers. **(a)** DNN. **(b)** LSTM. **(c)** Attention LSTM.

Figures 3a, 4a show that the DNN model trains very fast as its training accuracy hits 99% while its validation accuracy stays within the 99% range. The model successfully processed a range of inputs while maintaining high processing speed, making it a suitable option when speed is paramount and data types are straightforward. The straightforward training process and strong performance make this model effective for basic tasks. Our LSTM model in Figures 3b, 4b demonstrated a slow yet steady training improvement. It started with 71% validation accuracy and gradually increased to 98% over time. Despite a gradual learning pace, the Long Short-Term Memory achieved stable, progressive results during training because it processed sequential information effectively. The LSTM network achieves high accuracy on sequence data, enabling it to handle datasets that require modeling temporal dependencies. The Attention-based LSTM model in Figures 3c, 4c outperformed LSTM in both validation loss and generalization with minimal overfitting. The model trained more slowly than DNN and LSTM, yet attention mechanisms helped it focus on vital features, improving its performance on activities that require managing long-term relationships. The model achieves high accuracy and enhanced predictive performance across diverse situations, but its additional features may not always be necessary for straightforward tasks. The DNN performs well for quickly processing simple tasks. At the same time, the LSTM effectively handles sequential processes, and the Attention LSTM provides the best solution for analyzing tasks with extended time-based relations. The DNN model delivers the fastest results among these models, but performs worse than LSTM and Attention LSTM in complex applications.

**Figure 5:**
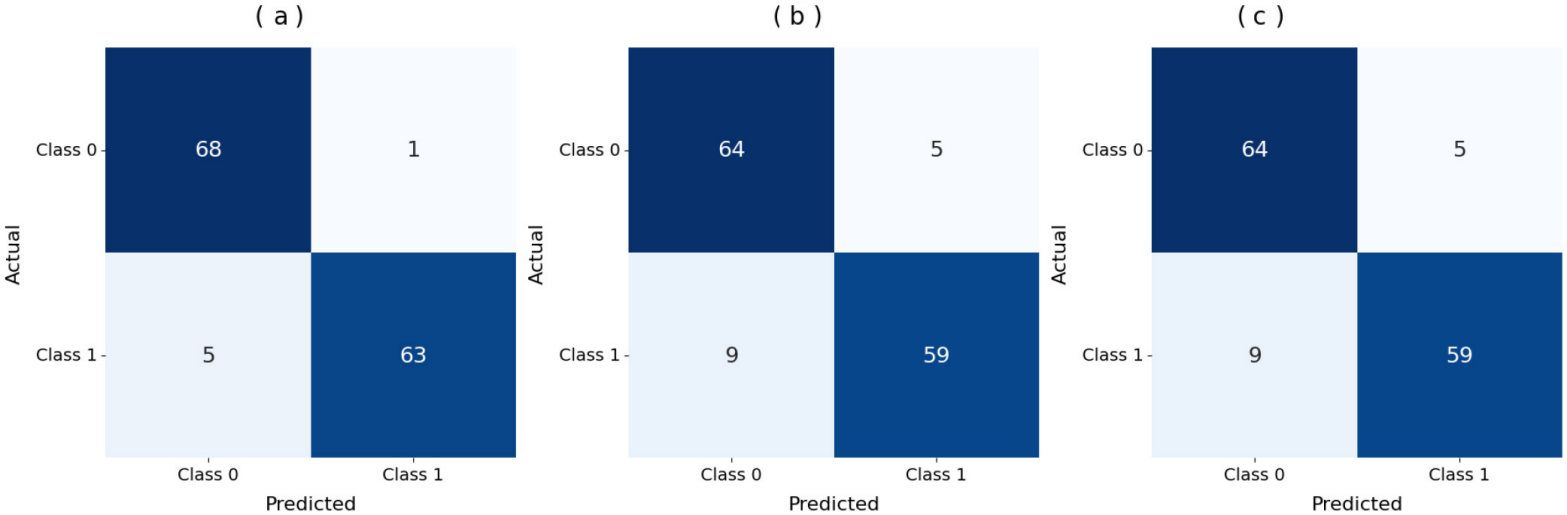
Confusion matrix of the models. **(a)** DNN. **(b)** LSTM. **(c)** Attention LSTM.

Figure 5a displays the DNN model correctly identified 68 positive samples but failed to recognize 5 instances that belonged to that class. The DNN model achieved good results but struggled to differentiate between classes, particularly in some cases. The results demonstrate effective performance, with a few incorrect instances across categories. When evaluating Figure 5b, the LSTM model labeled 64 cases as positive, although it got 9 instances wrong. The system correctly marked 59 samples as negative. The LSTM outperforms a DNN but still exhibits additional errors in identifying positive samples in this dataset. Figure 5c shows that the Attention-based LSTM model duplicated its confusion matrix results with the LSTM, classifying 64 positive instances correctly and misclassifying 9 of them while also correctly identifying 59 cases of the negative class. Fabulously, the attention mechanism performed on par with the simple LSTM model in this context. Both models correctly identify a substantial number of instances without significant performance reduction. All three systems perform well, but each misclassifies approximately the same number of cases. Although the three models perform similarly in classification, they differ in their internal designs. The DNN is the most stable model for this dataset, whereas the LSTM and Attention LSTM perform equally well on class identification despite the attention enhancements.

### 5.2 Transfer learning performance

Transfer Learning helped our models achieve better performance across multiple datasets. A pre-trained model can produce better results more quickly through transfer learning on new datasets. Researchers tested whether transfer learning improved model performance on Datasets 2 and 3 when unseen data were present. The model accurately detected Class 1 cases in Dataset 2, achieving a high recall of 99%, as shown in Table 5. The model shows reliable performance for both classes, generating very few false Class 0 and Class 1 predictions. The model accurately detected all instances labeled Class 0 and Class 1, with precision ratings of 0.97 for both classes. The model recovered most of the class cases, thereby reducing the number of undetected instances. Class 0 had a lower recall than class 1, yet still achieved an excellent F1 score of 0.95. The model achieved 97% accuracy on Dataset 2, demonstrating strong generalization to an unseen dataset. The drop to 92% on Dataset 3 reflects differences in symptom manifestation and feature distributions, highlighting inherent variability in cross-dataset implementation.

**Table 5:** Classification report for transfer learning model on datasets 2 and 3.

**Dataset**	**Class**	**Precision**	**Recall**	**F1-score**
Dataset 2	0	0.97	0.94	0.95
	1	0.97	0.99	0.98
Dataset 3	0	0.95	0.88	0.91
	1	0.88	0.96	0.92
Accuracy	-	-	-	0.97 (Dataset 2)
Accuracy	-	-	-	0.92 (Dataset 3)

This threshold-based performance analysis, presented in Table 6, demonstrates that the two models exhibit strong discriminative ability, with AUC scores of 0.9952 and 0.950, respectively. In the first model, the best threshold was 0.930, yielding a recall of 0.9855 and a specificity of 0.9394, providing an excellent balance between false positives and false negatives. Such an arrangement demonstrates the strength of this model and its high capacity to reduce false negatives without compromising true negative rates. Conversely, the second model had an optimal threshold of 0.677, achieving the highest recall (0.953) and specificity (0.880). Although its AUC is high at 0.950, the lower threshold and decreased specificity suggest that the model has a weak trade-off between sensitivity and precision, tending to yield positive results rather than absolute discrimination. Overall, both models were highly accurate, with the former offering a more favorable sensitivity-to-specificity ratio, making it more consistent and reproducible across different decision thresholds.

**Table 6:** Performance metrics comparison at optimal thresholds.

**Dataset**	**Optimal threshold**	**Sensitivity**	**Specificity**	**AUC**
Dataset 2	0.930	0.9855	0.9394	0.9952
Dataset 3	0.677	0.9530	0.8800	0.9500

In the case of Dataset 3 testing, the model showed more balanced performance than before. In Class 0, it had a precision of 0.95 and a recall of 0.88, yielding an F1 score of 0.91. This indicates that the model correctly identified most Class 0 samples, with only a few misclassifications. In Class 1, a model achieved a precision of 0.88 and a recall of 0.96, yielding an F1 score of 0.92. These findings indicate that the model performed better on Class 1 samples, with a minor overprediction bias. In total, the model scored 0.92 on Dataset 3, demonstrating competent but less stable performance compared with Dataset 2, which scored 0.97 and achieved nearly perfect class-wise balance. The difference indicates that the transfer-learning model was well generalized to Dataset 3. However, the dataset was slightly more challenging, resulting in differences in recognition consistency across classes.

**Figure 6:**
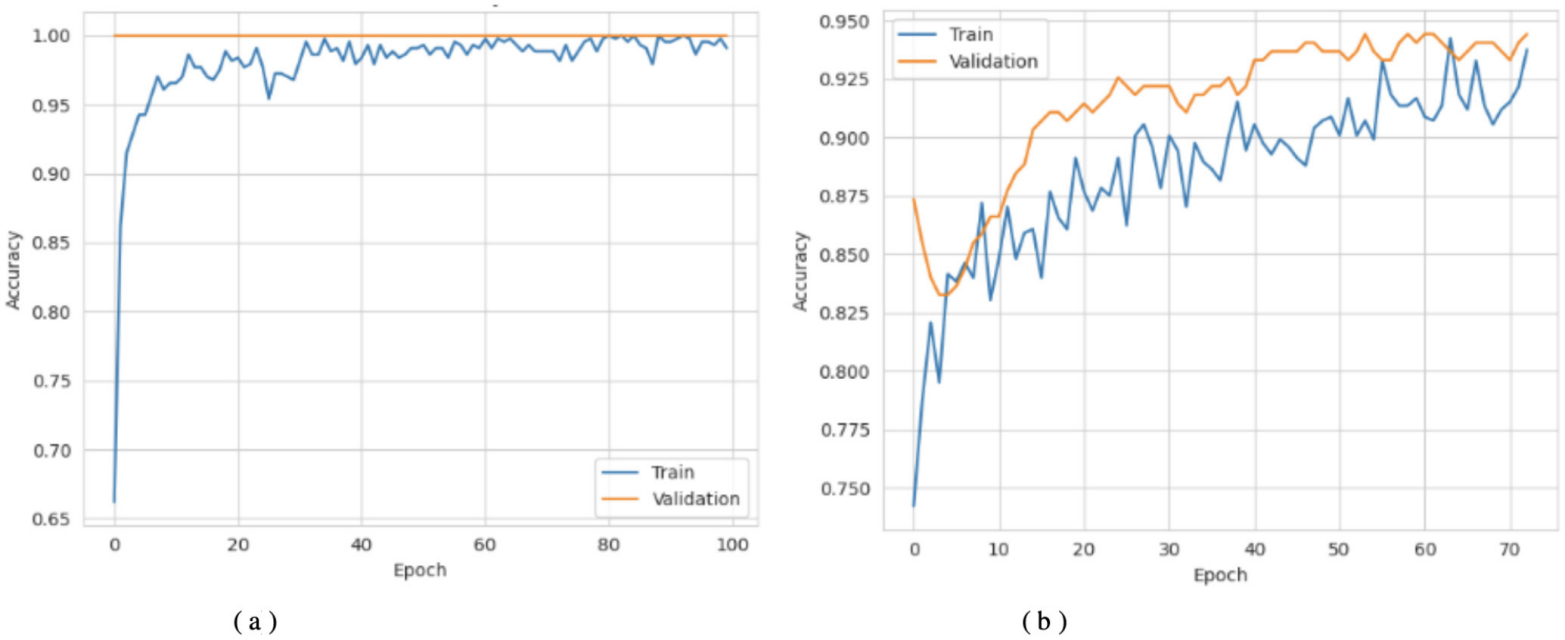
Accuracy curves of TL-DNN on datasets 2 and 3. **(a)** TL-DNN on dataset 2. **(b)** TL-DNN on dataset 3.

**Figure 7:**
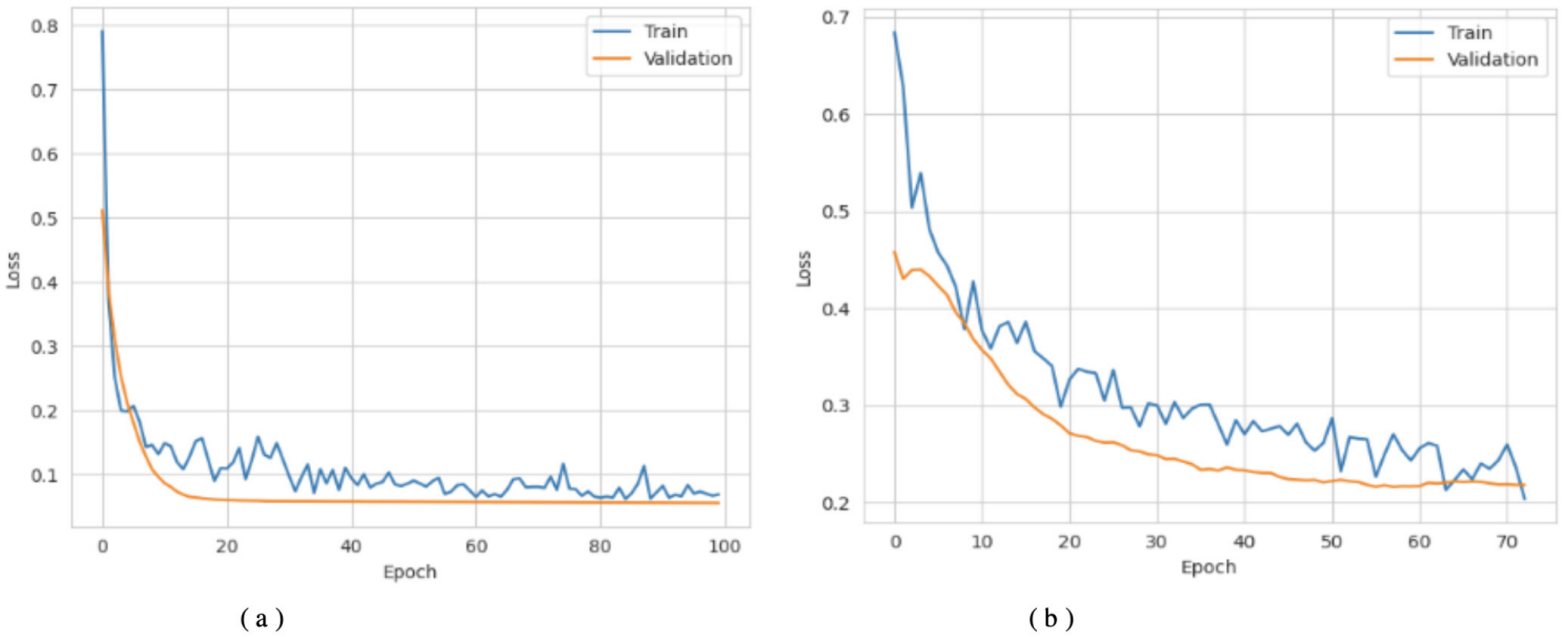
Loss curves of TL-DNN on datasets 2 and 3. **(a)** TL-DNN on dataset 2. **(b)** TL-DNN on dataset 3.

**Figure 8:**
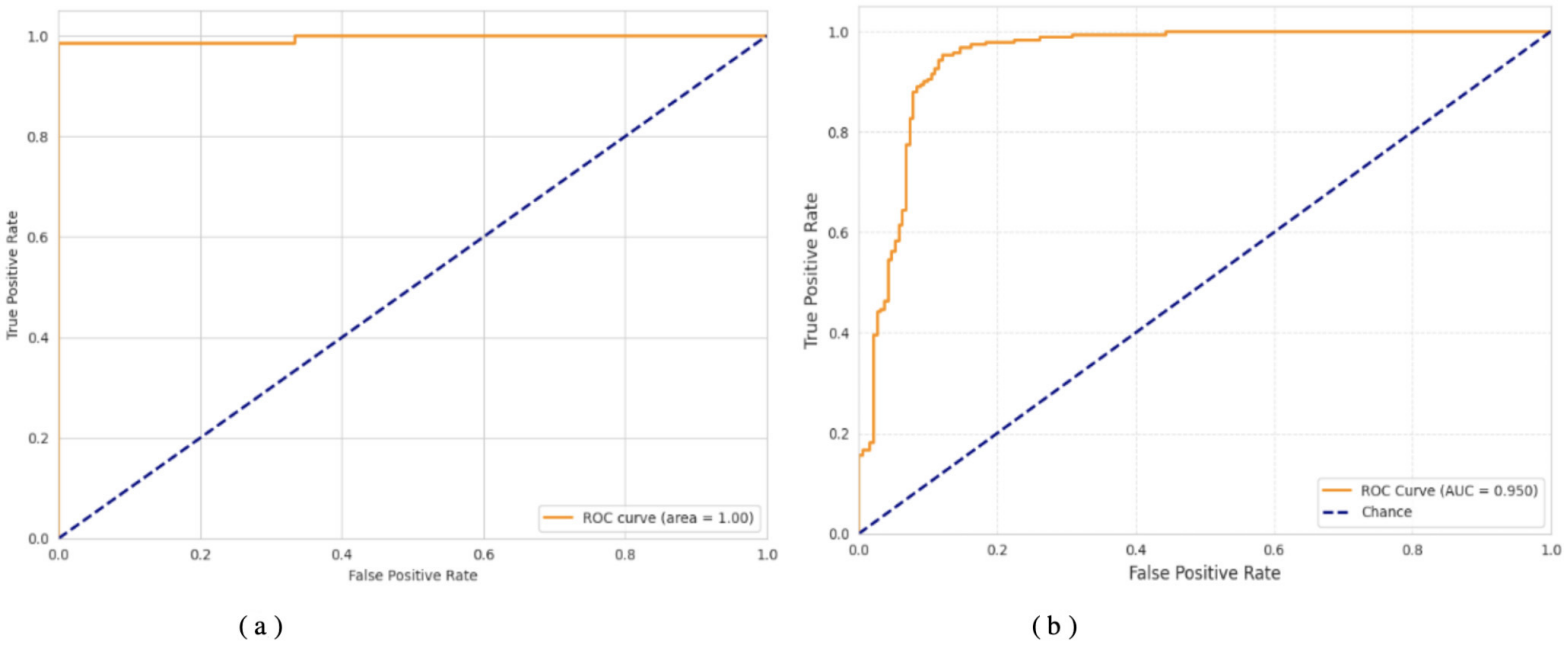
ROC curves of TL-DNN on datasets 2 and 3. **(a)** TL-DNN on dataset 2. **(b)** TL-DNN on dataset 3.

Figures 6–8 show how the Transfer Learning Deep Neural Network (TL-DNN) behaves during the learning phase and classifies data from Datasets 2 and 3. The chart in Figure 4 shows that TL-DNN delivers top-quality results on both datasets, with Dataset 2 showing rapid performance gains to near 100% accuracy. After an unstable start, Dataset 3 reaches a stable 95% validation accuracy by adapting to different data patterns. The results shown in Figure 7 support the observation of the training process. The training process remains stable, with validation and training losses declining rapidly on Dataset 2. The loss starts high but fluctuates frequently during early training before improving.

Figure 8 displays superior classification performance for both datasets, as shown by AUC results of 1.0 and 0.950, respectively. The results show that the model performs effectively across all decision thresholds, with high detection efficiency. TL-DNN produces reliable results across datasets, with robust performance on Dataset 2 and solid performance on Dataset 3.

**Figure 9:**
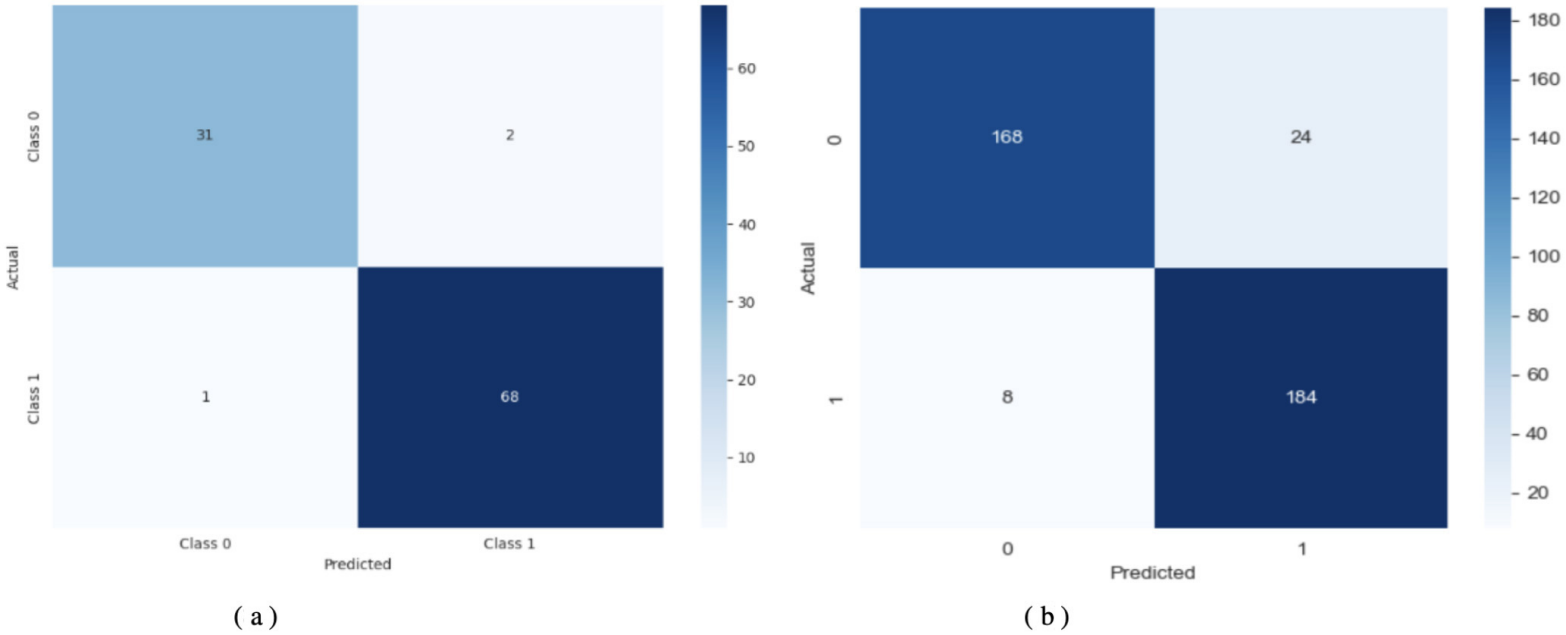
Confusion matrix of TL-DNN on datasets 2 and 3. **(a)** TL-DNN on dataset 2. **(b)** TL-DNN on dataset 3.

Figure 9 shows a detailed assessment of how the model classifies the data. Using TL-DNN on Dataset 2 yields effective predictions, with the model correctly identifying most instances, though a few errors remain. On Dataset 3, this model provides more precise matches, but makes relatively more false positives. The performance drop may result from the model failing to process matching features or from unequal class sample sizes.

### 5.3 XAI insights

The model's decisions were analyzed using XAI to improve interpretability. The analysis identified which features played the strongest role in shaping model output. The Shapley Additive Explanations (SHAP) method estimated the extent to which each feature contributed to the final results. The SHAP value is computed using Equation 13, which requires evaluating all subsets of features excluding the target feature. Consequently, the computational complexity grows exponentially with the number of features, i.e., *O*(2^|*N*|^), making exact computation intractable for high-dimensional datasets.


$$\begin{array}{l} \phi_{i}=\underset{S\subseteq N\backslash \left\{i\right\}}{\sum }\frac{|S|!\left(|N|-|S|-1\right)!}{|N|!}\left[f\left(S\cup \left\{i\right\}\right)-f\left(S\right)\right] \end{array}$$ (13)

**Figure 10:**
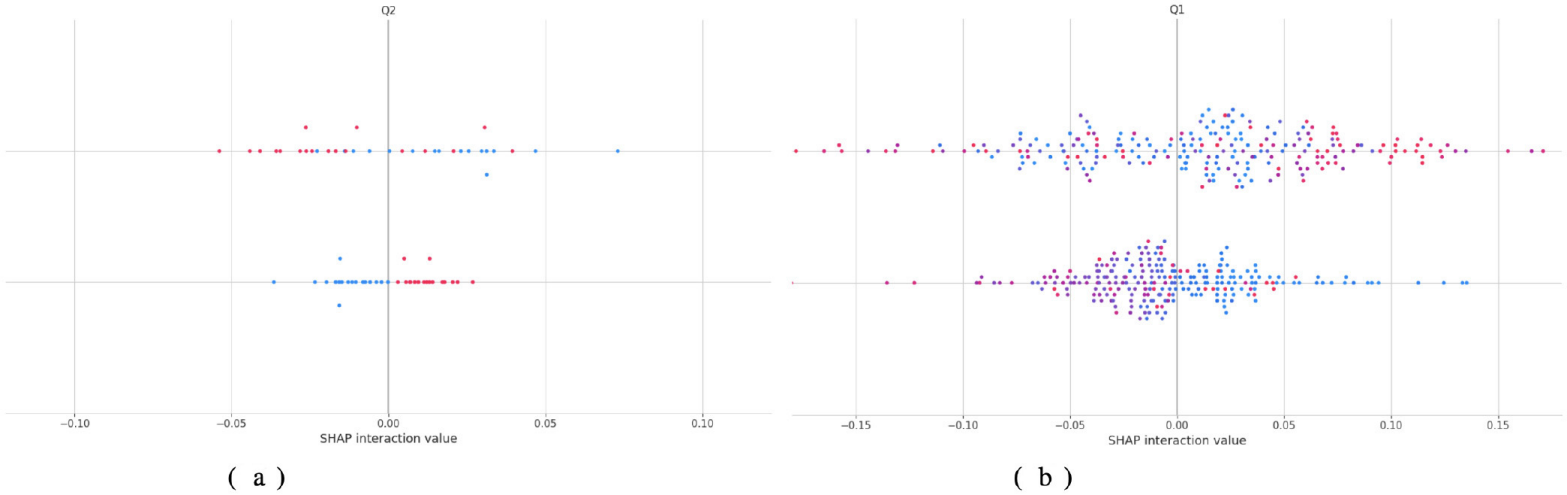
Shap analysis of TL-DNN on datasets 2 and 3. **(a)** TL-DNN on dataset 2. **(b)** TL-DNN on dataset 3.

Figure 10 describes the contribution of each feature and its interaction with others to the decisions of the TL-DNN model using SHAP interaction plots in Figure 2 below: one can observe that in Dataset 2, both single and combined contributions of features are significant, whereas in Dataset 3, feature contribution and feature interaction contribution are more balanced. In Dataset 2, the values of interaction are clustered near zero, revealing that the model is based more on the contribution of single features, as opposed to a higher-order association. This behavior is consistent with a dataset in which features exhibit restricted variability and weak cross-feature interactions. Conversely, Dataset 3 exhibits more compact, wider clusters of SHAP interaction values, particularly for feature Q1, indicating significant nonlinear interactions. These trends indicate that the TL-DNN adopts more complex reasoning when working with Dataset 3, perhaps because Dataset 3 exhibits richer behavioral differences.

**Figure 11:**
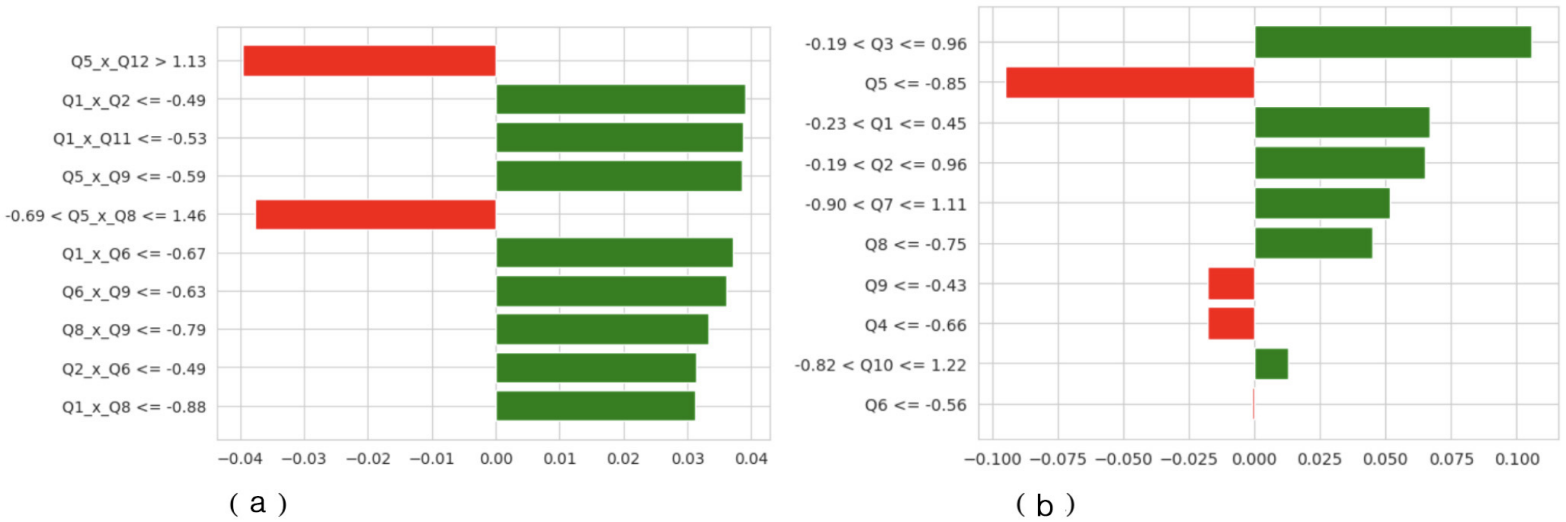
LIME of TL-DNN on datasets 2 and 3. **(a)** TL-DNN on dataset 2. **(b)** TL-DNN on dataset 3. X-axis shows LIME Value and Y-axis shows Labels.

An additional explanation is in Figure 11 by attributing features of LIME. In Dataset 2, the relative proportions of positive and negative contributions are approximately equal, indicating that no particular behavioral characteristic strongly influences the model's predictions. Specific behavioral differences used as decision pivots are captured by threshold-based rules such as *Q*5*xQ*12 > 1.13 and *Q*1*xQ*2 = −0.49. In Dataset 3, the distribution is more skewed, and the features that have the most impact, like Q5, Q3, and Q1, have the most positive and negative effects. The bar lengths of the LIME plots indicate that predictions in Dataset 3 are highly reliant on these behavioral indicators, as was observed in the more complex SHAP interactions above. Combined, the results from SHAP and LIME support the finding that the TL-DNN model is deep and clinically consistent in pattern recognition when trained on a richly featured dataset.

A number of the most significant effects detected when using both SHAP and LIME are those that can be linked to constructs that are common in ASD screening models. On the one hand, characteristics such as Q1, Q3, and Q5 that predominate in the interaction patterns of SHAP in Dataset 3 are associated with social reciprocity, the use of communication cues, and repetitive behaviors. The domains are consistent with the basic diagnostic criteria of the most popular ASD assessment instruments, the M-CHAT and the ADOS. They dominate the model's inner logic and therefore indicate that the TL-DNN is learning and understanding clinically significant behavioral abnormalities, rather than relying on noise or irrelevant patterns. Similarly, cross-term relationships in Dataset 2 (Q5 × Q12 and Q1 × Q2) indicate that combinations of behaviors (e.g., joint-attention cues and communication responses) can affect classification outcomes, supporting the idea that the model focuses on relationships consistent with real-world co-occurrence patterns among behaviors.

The interpretation indicates that TL-DNN connects inputs more freely in Dataset 2 but forms deeper associations in Dataset 3. The TL-DNN model demonstrates adequate data flexibility, and its feature identification increases people's trust in its predictions. The TL model achieved an excellent 91% accuracy on Dataset 2, but produced less balanced results on Dataset 3. When classifying Class 1 records, the model produced several false positives, reducing precision to 0.58 while maintaining good scores for Class 0. Despite better retrieval of Class 1 data, the model achieved 84% accuracy.

## 6 Discussion

The test results demonstrate the benefits of transfer learning in healthcare settings. Our analysis evaluated the utility of the DNN-based model by assessing accuracy and precision, recall, and F1-score across multiple datasets. The TL model achieved an excellent 91% accuracy on Dataset 2. Based on our analysis, the TL model detected 100% of the real instances from Class 0. In the Class 1 classification, this approach accurately identified all matching results, achieving an F1-score of 0.92. The model successfully predicted new data, correctly classifying between the two classes with few errors. The model generated less balanced results on Dataset 3. When classifying Class 1 records, the model produced several false positives, reducing precision to 0.58 while maintaining good scores for Class 0. Despite improved retrieval of Class 1 data, the model achieved 84% accuracy because it found Dataset 3 to be too unbalanced for effective performance. The system struggles to detect members of the smallest class (Class 1) while achieving good results with Class 0 cases.

The ability of these models to properly label clinical patients can lead to significant improvements in patient outcomes. For Dataset 2, the high-quality results make the model suitable for medical use in diagnostic tools and condition identification. The model provides reliable diagnostic support through its spot-on recognition of Class 0 and strong measurement accuracy for Class 1. Our model performed worse on dataset 3, particularly for class 1, because the data were imbalanced and included items that were hard to recognize. To address these cases, the system requires adjustments, such as balancing the dataset or further training the model. The model's performance across various datasets indicates that medical practice requires training tailored to each patient setting.

The findings from this research support similar projects that apply transfer learning in medical and diagnostic work. The effectiveness of transfer learning techniques has been established in previous studies, particularly in datasets with limited labeling and in medical image recognition applications (e.g., X-ray and MRI scans). This study contributes to the evidence on the effectiveness of transfer learning methods in practical classification problems and highlights that models require appropriate adjustments to account for dataset characteristics. The difference in model performance stems from typical machine learning issues. Models perform well on specific datasets but struggle with variations in data quality, feature attributes, or class distributions. The research findings from this work align with previous studies documenting challenges in analyzing complex datasets, highlighting the need to develop specialized methods and maintain active model development.

In the article by (14), pre-trained deep CNN with hybrid machine learning classifiers (SqueezeNet + SVM, KNN and DT) were employed to classify the severity of ASD and make a distinction between ASD and control subjects with the highest possible accuracy of 87.8%. Although their effort demonstrated the feasibility of EEG-based transfer learning and hybrid modeling, the work was limited to single-data testing and was not interpretable. The Deep Transfer Learning and Explainable AI-based system achieves 97% accuracy, enabled by multi-dataset transfer learning, irrespective of age or demographic factors. In addition, a combination of Grad-CAM and SHAP increases transparency by visualizing the most significant neuro-behavioral aspects; hence, performance across diverse datasets and at the clinical level is superior to that of other methods.

The current method is promising, but it has several limitations in its operation. There is an essential limitation of the approach: it relies on previously trained models that may perform less well on datasets with significantly different distribution patterns or characteristics. According to Dataset 3, the model requires modification by incorporating strategies such as oversampling and undersampling, as well as specialized loss functions, to achieve improved results in Class 1 classification. The models are overfitting, which yields good performance on the training data but leads to poor performance on new, unseen data points. The advantage of transfer learning with pre-trained features does not preclude the need for model adaptation and testing on new data. The current research assessed only accuracy and simple classification performance measures. Future research should give serious consideration to cross-validation, as it would help evaluate the model's strength across varied datasets and test its performance under different conditions.

Despite the high rate of performance implemented by the proposed framework, there are some serious problems in terms of translating learning implementation to ASD Classification relying on behavioral data. Heterogeneity among the datasets is the primary issue, as the three datasets have different demographic structures, feature sets, and data-collection methods. Such differences complicate the concept of smooth knowledge transfer and the identification of feature similarities across domains. Moreover, data imbalance and the absence of behavioral traits can impede model convergence and degrade generalization to other datasets. The other weakness is that there are no standard diagnostic and annotation protocols that may lead to biasness in model adaptation. To address the issues, more complicated domain-adaptation, feature-alignment, and harmonization strategies will be required to obtain robust, transferable learning in different datasets of ASD.

The weakness of the work is that the datasets used are heterogeneous in development. ASD behaviors in toddlers are significantly different from those in adolescents and adults in the way that they are manifested and in the way that they are diagnosed. Consequently, one should not draw sweeping conclusions of inter-age applicability. The observed model's performance does not imply that there are common behavioral indicators across such populations; it merely suggests that certain questionnaire-response patterns can be generalized across instruments. The differences should be better accounted for in future work using age-stratified datasets or developmental normalization techniques.

According to the current findings, several solutions would lead to an even more precise, generalized and practical framework. To increase the representativeness of the analysis and to focus on larger multicenter behavioral samples, it is recommended that the analysis be expanded to these samples. Other behavioral measures, such as facial expression analysis, speech patterns, and caregiver reports, can be modulated to yield more specific information for determining ASD. Furthermore, data augmentation and adaptive feature selection procedures can help address class imbalance and small-sample learning. Finally, collaboration between institutions could be established through federated learning and privacy-preserving training methods, while ensuring the security of sensitive health data, thereby facilitating the scalable and ethically responsible application of such solutions in real-world clinical contexts.

## 7 Conclusion and future work

This paper presented a transfer-learning approach for ASD Classification using Deep Neural Networks (DNN) across three distinct datasets. This study demonstrated that a pre-trained deep neural network, applied with transfer learning, showed strong promise for classifying medical datasets. The model achieved excellent results when trained on Dataset 2; however, its performance decreased on Dataset 3 due to the dataset's unbalanced class distribution. The models demonstrate a robust capacity to apply knowledge gained from one dataset to new datasets with scarce labeled examples for medical diagnosis. When used, the TL model enhances diagnostic accuracy in settings with scarce clinical evidence. Because it can transfer knowledge across datasets, the technique is helpful in medical facilities operating with limited labeled data. The results on Dataset 3 demonstrate that additional methods are necessary to handle imbalanced data effectively, enabling a more robust practical implementation. Although the proposed framework has strong potential for identifying early behavioral patterns associated with ASD, it has limitations. The self-reported questionnaire data used in the current study were obtained through guardians. While they are helpful for early-stage mass screening, they are subject to bias and have not been clinically validated. Nevertheless, since the model's primary objective is to facilitate early detection rather than diagnosis, it remains suitable for identifying behavioral inclinations that warrant referral to the next professional level. The explainability evaluation using SHAP and LIME successfully highlights the most critical behavioral and developmental characteristics that inform the classification of outcomes. However, these meanings are limited to data-supported insights and do not yet incorporate clinical judgment or analysis of misclassification. Future work will aim to address this gap by collaborating with clinicians to confirm interpretability results and explore model behavior in borderline or ambiguous cases. Future research will address the current shortcomings by testing the framework on larger, multicentre datasets to enhance generalizability. Clinical understanding could be improved by incorporating multimodal neuroimaging (MRI, fNIRS, eye-tracking, and EEG) and wearable devices. Federated and privacy-preserving learning will enable collaborative model training without compromising patient data. Adaptive continuous learning may incorporate new clinical information to enhance diagnostic accuracy. Lastly, the explainable human-AI interfaces will allow clinicians to view the reason why the model made its decisions, which will assist in early intervention and more reliable neurodevelopmental diagnosis.

## Data Availability

The original contributions presented in the study are included in the article/supplementary material, further inquiries can be directed to the corresponding author.

## Footnotes

1
https://www.kaggle.com/datasets/asdpredictioninsaudi/asd-screening-data-for-toddlers-in-saudi-arabia

2
https://www.scidb.cn/en/detail?dataSetId=0b84f15557744486a2d59366716d4f8d

## References

[1] Almadhor A Alasiry A Alsubai S Al Hejaili A Kovac U Abbas S. Explainable and secure framework for autism prediction using multimodal eye tracking and kinematic data. Complex Intellig Syst. (2025) 11:173. doi: 10.1007/s40747-025-01790-3

[2] Bertelli MO Azeem MW Underwood L Scattoni ML Persico AM Ricciardello A . Autism spectrum disorder. In: Textbook of Psychiatry for Intellectual Disability and Autism Spectrum Disorder. Cham: Springer (2022). p. 369–455.

[3] Wang M Zhang X Zhong L Zeng L Li L Yao P. Understanding autism: causes, diagnosis, and advancing therapies. Brain Res Bullet. (2025) 227:111411. doi: 10.1016/j.brainresbull.2025.111411 40449388

[4] Qin L Wang H Ning W Cui M Wang Q. New advances in the diagnosis and treatment of autism spectrum disorders. Eur J Med Res. (2024) 29:322. doi: 10.1186/s40001-024-01916-2 38858682 PMC11163702

[5] Lee J Lee TS Lee S Jang J Yoo S Choi Y . Development and application of a metaverse-based social skills training program for children with autism spectrum disorder to improve social interaction: protocol for a randomized controlled trial. JMIR Res Protoc. (2022) 11:e35960. doi: 10.2196/35960 35675112 PMC9218883

[6] Atlam ES Aljuhani KO Gad I Abdelrahim EM Atwa AEM Ahmed A. Automated identification of autism spectrum disorder from facial images using explainable deep learning models. Sci Rep. (2025) 15:26682. doi: 10.1038/s41598-025-11847-5 40695996 PMC12283938

[7] Rashed AEE Bahgat WM Ahmed A Farrag TA Atwa AEM. Efficient machine learning models across multiple datasets for autism spectrum disorder diagnoses. Biomed Signal Proc Cont. (2025) 100:106949. doi: 10.1016/j.bspc.2024.106949

[8] Rubio-Martín S García-Ordás MT Bayón-Gutiérrez M Prieto-Fernández N Benítez-Andrades JA. Enhancing ASD detection accuracy: a combined approach of machine learning and deep learning models with natural language processing. Health Inform Sci Syst. (2024) 12:20. doi: 10.1007/s13755-024-00281-y 38455725 PMC10917721

[9] Joudar SS Albahri AS Hamid RA Zahid IA Alqaysi ME Albahri OS . Artificial intelligence-based approaches for improving the diagnosis, triage, and prioritization of autism spectrum disorder: a systematic review of current trends and open issues. Artif Intellig Rev. (2023) 56:53–117. doi: 10.1007/s10462-023-10536-x

[10] Sui J Jiang R Bustillo J Calhoun V. Neuroimaging-based individualized prediction of cognition and behavior for mental disorders and health: methods and promises. Biol Psychiatry. (2020) 88:818–28. doi: 10.1016/j.biopsych.2020.02.016 32336400 PMC7483317

[11] Alam M Rashid M Ali M Yvette S. Addressing the challenge of dataset acquisition for ASD diagnosis with deep learning-based neural networks. In: AIP Conference Proceedings. Melville, NY: AIP Publishing (2024).

[12] Abdelhamid N Padmavathy A Peebles D Thabtah F Goulder-Horobin D. Data imbalance in autism pre-diagnosis classification systems: an experimental study. J Inform Knowl Managem. (2020) 19:2040014. doi: 10.1142/S0219649220400146

[13] Al-Qazzaz NK Ali SHBM Ahmad SA. Quantifying autism spectrum disorder severity through multi-channel EEG-based entropy analysis. In: International Conference for Innovation in Biomedical Engineering and Life Sciences. Cham: Springer (2024). p. 154–162.

[14] Al-Qazzaz NK Aldoori AA Buniya AK Ali SHBM Ahmad SA. Transfer learning and hybrid deep convolutional neural networks models for autism spectrum disorder classification from EEG signals. IEEE Access. (2024) 12:64510–30. doi: 10.1109/ACCESS.2024.3396869

[15] Kumar CJ Das PR. The diagnosis of ASD using multiple machine learning techniques. Int J Dev Disabil. (2022) 68:973–83. doi: 10.1080/20473869.2021.1933730 36568623 PMC9788716

[16] Ghazal TM Munir S Abbas S Athar A Alrababah H Khan MA. Early detection of autism in children using transfer learning. Intellig Autom Soft Comp. (2023) 36:11–22. doi: 10.32604/iasc.2023.030125

[17] Ashraf A Qingjie Z Bangyal WHK Iqbal M. Analysis of brain imaging data for the detection of early age autism spectrum disorder using transfer learning approaches for internet of things. IEEE Trans Consumer Elect. (2023) 70:4478–89. doi: 10.1109/TCE.2023.3328479

[18] Herath L Meedeniya D Marasinghe J Weerasinghe V Tan T. Autism spectrum disorder identification using multi-model deep ensemble classifier with transfer learning. Expert Syst. (2025) 42:e13623. doi: 10.1111/exsy.13623

[19] Das A Alam T Hossain MZ Lamiya HR. Autism disease prediction using deep learning & transfer learning. In: 2024 2nd International Conference on Networking, Embedded and Wireless Systems (ICNEWS). Bangalore: IEEE (2024). p. 1–8.

[20] Kasri W Himeur Y Copiaco A Mansoor W Albanna A Eapen V. Hybrid vision transformer-mamba framework for autism diagnosis via eye-tracking analysis. arXiv [preprint] arXiv:250606886. (2025). doi: 10.1109/CCNCPS66785.2025.11135843

[21] Malik W Fahiem MA Farhat T Alghazo R Mahmood A Alhajlah M. An explainable deep learning framework for multimodal autism diagnosis using XAI GAMI-Net and hypernetworks. Diagnostics. (2025) 15:2232. doi: 10.3390/diagnostics15172232 40941719 PMC12427627

[22] Rakotomanana H Rouhafzay G A. Scoping review of ai-based approaches for detecting autism traits using voice and behavioral data. Bioengineering. (2025) 12:1136. doi: 10.3390/bioengineering12111136 41301092 PMC12649475

[23] Hatim HA Alyasseri ZAA Jamil N. A recent advances on autism spectrum disorders in diagnosing based on machine learning and deep learning. Artif Intellig Rev. (2025) 58:313. doi: 10.1007/s10462-025-11302-x

[24] Rajagopalan SS Zhang Y Yahia A Tammimies K. Machine learning prediction of autism spectrum disorder from a minimal set of medical and background information. JAMA Network Open. (2024) 7:e2429229. doi: 10.1001/jamanetworkopen.2024.29229 39158907 PMC11333987

[25] Sarwani IS Bhaskari DL Bhamidipati S. Emotion-based autism spectrum disorder detection by leveraging transfer learning and machine learning algorithms. Int J Adv Comp Sci Appl. (2024) 15:150556. doi: 10.14569/IJACSA.2024.0150556

[26] Singh S Malhotra D Mengi M. TransLearning ASD: Detection of autism spectrum disorder using domain adaptation and transfer learning-based approach on RS-FMRI data. In: Artificial Intelligence and Communication Technologies. New Delhi: SCRS (Soft Computing Research Society) (2023). p. 863–71.

[27] Seu K Kang MS Lee H. An intelligent missing data imputation techniques: a review. JOIV: Int J Inform Visualizat. (2022) 6:278–83. doi: 10.30630/joiv.6.1-2.935

[28] Cheng H Zhang M Shi JQ. A survey on deep neural network pruning: Taxonomy, comparison, analysis, and recommendations. IEEE Trans Pattern Anal Mach Intell. (2024). 46:10558–78. doi: 10.1109/TPAMI.2024.3447085 39167504

[29] Iman M Arabnia HR Rasheed K A. review of deep transfer learning and recent advancements. Technologies. (2023) 11:40. doi: 10.3390/technologies11020040

